# Curcumin-preconditioned bone marrow mesenchymal stem cell exosomes ameliorate postmenopausal osteoporosis by activating mitophagy in osteoblast-lineage cells

**DOI:** 10.3389/fcell.2026.1891247

**Published:** 2026-07-29

**Authors:** Chao Li, Xufeng Jiang, Kechong Zhou, Quan Sun, Huaqing Shen

**Affiliations:** 1 Department of Orthopedics, Xiangyang Central Hospital, Affiliated Hospital of Hubei University of Arts and Science, Xiangyang, Hubei, China; 2 Department of Urology, Xiangyang Central Hospital, Affiliated Hospital of Hubei University of Arts and Science, Xiangyang, Hubei, China

**Keywords:** curcumin, mesenchymal stem cell exosomes, mitophagy, PINK1/parkin pathway, postmenopausal osteoporosis, single-cell RNA sequencing

## Abstract

**Background:**

Postmenopausal osteoporosis, driven by oxidative stress and impaired osteoblast function, remains a major health crisis with limited anabolic therapies. Exosomes from curcumin-preconditioned mesenchymal stem cells (MSCs) are promising cell-free therapeutics, yet their cellular targets and molecular mechanisms remain elusive.

**Methods:**

BMSCs were isolated, pretreated with curcumin, and exosomes were purified by ultracentrifugation. Exploratory scRNA-seq, *in vitro* H_2_O_2_ stress assays, and ovariectomized (OVX) mouse models were employed.

**Results:**

Curcumin pretreatment enhanced exosomal osteogenic potential without altering physicochemical properties. scRNA-seq identified osteoblast-lineage cells as major transcriptional responders. Cur-BMSC-Exo activated PINK1/Parkin-mediated mitophagy, eliminated damaged mitochondria, and restored mitochondrial function. This effect was abrogated by Mdivi1. Systemic Cur-BMSC-Exo administration significantly increased bone volume and trabecular thickness in OVX mice, whereas Mdivi1 abolished these benefits.

**Conclusion:**

Cur-BMSC-Exo ameliorates osteoporosis by activating mitophagy in osteoblast-lineage cells, offering a novel cell-free therapeutic strategy.

## 1 Background

Postmenopausal osteoporosis, characterized by excessive bone resorption and impaired bone formation, afflicts more than 200 million women globally, constituting a substantial public health challenge linked to elevated fracture susceptibility, considerable disability, and increased mortality (Black and Rosen, 2016). Currently, there is an exigent need to develop novel, safe, and effective anabolic therapies that promote osteoblast-mediated bone formation (Altkorn and Vokes, 2001; Appelman-Dijkstra and Papapoulos, 2015). As a promising acellular therapeutic option for bone regeneration, mesenchymal stem cell-derived exosomes possess multiple advantages over cell-based therapies, most notably their low immunogenicity, high biocompatibility, and minimal risk of tumorigenesis (Appelman-Dijkstra and Papapoulos, 2015; Huang et al., 2018). Exosomes are nanosized extracellular vesicles that carry bioactive molecules such as proteins, lipids, and nucleic acids, mediating intercellular communication and regulating various biological processes (Tian et al., 2023; Wang et al., 2023). However, the therapeutic efficacy of native MSC-derived exosomes is often limited by their relatively low potency and short half-life *in vivo* (Riazifar et al., 2019).

Curcumin, a natural polyphenol derived from the rhizome of *Curcuma longa*, has been shown to possess potent anti-inflammatory, antioxidant, and osteogenic properties. However, the clinical translation of native curcumin is critically impeded by its inherent poor aqueous solubility, limited systemic bioavailability, and rapid metabolic clearance. Recent studies have demonstrated that preconditioning MSCs with curcumin can significantly enhance the therapeutic potential of their secreted exosomes by modulating their cargo content, providing an effective strategy to improve curcumin delivery and efficacy (Tawfeek and Kasem, 2023). Curcumin pretreatment reprograms the transcriptomic and proteomic profile, leading to the enrichment of specific bioactive cargoes in exosomes. For example, curcumin-preconditioned mesenchymal stem cells exosomes have been shown to carry higher levels of antioxidant enzymes, anti-inflammatory microRNAs compared with native exosomes (Tawfeek and Kasem, 2023). Importantly, emerging evidence indicates that curcumin exerts chondroprotective effects in osteoarthritis by reducing mitochondrial ROS production, enhancing mitochondrial biogenesis, and promoting mitophagy (Jin et al., 2022). Concurrently, exosomes can activate PINK1/Parkin-dependent mitophagy in recipient cells to restore mitochondrial homeostasis and alleviate cellular injury (Li Y. et al., 2024). This mitochondrial reprogramming effect is particularly relevant for bone regeneration, as osteoblasts are highly metabolically active cells that depend on intact mitochondrial function for energy production and differentiation.

Oxidative stress plays a central role in the pathogenesis of postmenopausal osteoporosis, as estrogen deficiency leads to excessive ROS production, which impairs osteoblast differentiation and induces osteoblast apoptosis (Iantomasi et al., 2023; Luo et al., 2025; Zhao et al., 2021). Mitochondria are the primary source of ROS in osteoblasts, and dysfunctional mitochondria further amplify oxidative stress, perpetuating a vicious cycle that compromises bone homeostasis. As a selective autophagic process that recognizes and eliminates dysfunctional mitochondria, mitophagy plays a critical role in preserving mitochondrial integrity and safeguarding intracellular redox homeostasis (Wang et al., 2020). Emerging evidence suggests that dysregulated mitophagy contributes to the development of osteoporosis, and enhancing mitophagy may represent a novel therapeutic approach to promote bone formation (Zeng et al., 2022). Despite these advances, the cellular and molecular mechanisms by which curcumin-preconditioned BMSC-derived exosomes (Cur-BMSC-Exo) exert their osteogenic effects remain poorly understood. In particular, whether Cur-BMSC-Exo exerts its therapeutic effects by modulating mitophagy in osteoblast-lineage cells has not been investigated. Furthermore, the specific cell types targeted by Cur-BMSC-Exo *in vivo* and the transcriptional changes induced by these exosomes at the single-cell level remain largely unknown.

In this study, we aimed to investigate the therapeutic potential of Cur-BMSC-Exo for postmenopausal osteoporosis and elucidate its underlying mechanisms. We first characterized BMSCs and their derived exosomes with or without curcumin pretreatment, and evaluated their biocompatibility, cellular uptake, and *in vivo* biodistribution. We then performed single-cell RNA sequencing (scRNA-seq) to identify the primary cellular target of Cur-BMSC-Exo in bone tissue and map the transcriptional changes induced by these exosomes. Finally, we validated our findings using *in vitro* oxidative stress models and *in vivo* ovariectomized (OVX) mouse models, combined with the specific mitophagy inhibitor Mdivi1. Our results demonstrate that Cur-BMSC-Exo preferentially targets osteoblast-lineage cells *in vivo*, activates PINK1/Parkin-mediated mitophagy, and enhances osteogenic differentiation, thereby ameliorating OVX-induced osteoporosis. This study provides novel insights into the mechanism of action of Cur-BMSC-Exo and supports its potential as a promising cell-free therapeutic strategy for postmenopausal osteoporosis.

## 2 Methods

### 2.1 Experimental animals

Prior to the commencement of any animal experiments, ethical approval was obtained from the Institutional Animal Care and Use Committee (IACUC) at Xiangyang Central Hospital. In accordance with the National Institutes of Health Guide for the Care and Use of Laboratory Animals, all experimental procedures were carried out appropriately. Female C57BL/6 mice, aged 6–8 weeks and weighing 18–22 g, were acquired from the Laboratory Animal Center of Xiangyang Central Hospital. The animals were maintained in a specific pathogen-free (SPF) facility under a 12-h light/dark cycle at a controlled ambient temperature of 22 °C ± 2 °C, with *ad libitum* access to standard laboratory chow and drinking water.

### 2.2 Isolation, culture and characterization of bone marrow-derived mesenchymal stem cells

We harvested bone marrow–derived mesenchymal stem cells from the femurs and tibias of 4-week-old male C57BL/6 mice using a previously reported method (Pittenger et al., 1999; Dominici et al., 2006). The marrow plugs were flushed out with α-MEM (#Gibco, USA) supplemented with 100 μg/mL streptomycin, 10% FBS (Gibco, USA) and100 U/mL penicillin, then dissociated into a single-cell suspension by filtering through a 70-μm cell strainer. Cells were plated in 10-cm dishes and maintained at 37 °C in a humidified incubator equilibrated with 5% CO_2_. Floating cells were removed at 48 h post-seeding, and fresh complete medium was provided at 3-day intervals. Experiments were conducted with cells at passage numbers 3 through 5.

Immunophenotyping of BMSCs was accomplished by labeling harvested cells with a panel of fluorescence-tagged antibodies — anti-CD29-APC, anti-CD34-PE, anti-CD44-FITC, anti-CD45-PE, anti-CD90-APC, and anti-CD105-FITC (BD Biosciences, USA) — for 30 min at 4 °C in the absence of light. Isotype controls were included in each run to establish background fluorescence thresholds. Cellular fluorescence was measured on a BD FACSCanto II instrument (BD Biosciences, USA), and population gating and statistical analysis were executed with FlowJo v10.8.1 (Tree Star, USA).

### 2.3 Curcumin pretreatment of BMSCs

A 10 mM curcumin (Sigma-Aldrich, USA) stock solution was reconstituted in dimethyl sulfoxide (DMSO; Sigma-Aldrich, USA), aliquoted, and maintained at −20 °C shielded from light. BMSCs were plated in 6-well tissue culture plates at a seeding density of 1 × 10^5^ cells per well and allowed to grow until reaching 70%–80% confluence. Thereafter, cells were exposed to graded concentrations of curcumin (0, 1.25, 2.5, 5, 10, 20, and 40 μM) for a 24-h period. Cell viability was quantified using the Cell Counting Kit-8 assay (CCK-8; Dojindo, Japan) following the manufacturer’s protocol, with optical density read at 450 nm on a microplate reader (Thermo Fisher Scientific, USA). The concentration of 10 μM, which yielded the highest cell viability while remaining within the non-toxic range, was selected as the preconditioning dose for all downstream experiments. This concentration was chosen to ensure biocompatibility of the preconditioning environment rather than to define an optimal osteogenic dose of curcumin itself.

### 2.4 Isolation and characterization of BMSC-derived exosomes

Exosomes were purified from the conditioned medium of untreated and curcumin-preconditioned BMSCs via sequential differential ultracentrifugation. The identity and purity of the isolated exosome preparations were confirmed by immunoblotting analysis of the exosome-specific markers CD9, CD63, and TSG101, and the absence of the endoplasmic reticulum contaminant Calnexin. Comprehensive protocols for exosome isolation and Western blotting are available in the Supplementary Material.

#### 2.4.1 Exosomal cargo characterization

To compare the cargo composition between BMSC-Exo and Cur-BMSC-Exo, total protein was extracted from purified exosomes (n = 3 independent batches per group) using RIPA lysis buffer supplemented with protease and phosphatase inhibitors. Protein concentration was determined by the BCA assay. Equal amounts of protein (20 μg per lane) were separated by 10% SDS-PAGE and transferred to PVDF membranes. After blocking with 5% non-fat milk, membranes were incubated overnight at 4 °C with primary antibodies against PINK1 (1:1,000; Abcam, ab23707) and CD63 (1:1,000; Santa Cruz Biotechnology, sc-5275), the latter serving as the exosomal loading control. Immunoreactive bands were visualized using enhanced chemiluminescence (ECL) and quantified by densitometry using ImageJ software (version 1.53k). Representative blot images and full quantitative data are provided in Supplementary Figure S1.

### 2.5 *In vitro* cellular uptake of exosomes

To investigate the internalization of exosomes by BMSCs, exosomal membranes were fluorescently labeled with the lipophilic carbocyanine dye DiI. Flow cytometry was performed to quantify the cellular uptake efficiency of native BMSC exosomes and Cur-BMSC-Exos, while confocal microscopy was used to visualize the intracellular localization of internalized exosomes. Full protocols are available in the Supplementary Material.

### 2.6 *In vivo* biodistribution of exosomes

For biodistribution assessment, DiI-tagged exosomes (200 μg suspended in 200 μL PBS) were delivered via tail vein injection to 8-week-old female C57BL/6 mice. Control animals received an equivalent volume of PBS alone. Twenty-four hours following administration, mice were rendered unconscious under isoflurane anesthesia and subjected to whole-body fluorescence scanning on an IVIS Spectrum imaging platform (PerkinElmer, USA). Subsequently, the animals were euthanized, and the heart, liver, spleen, lungs, and kidneys were excised for *ex vivo* fluorescence imaging. Signal quantification was performed with Living Image software (version 4.5.2; PerkinElmer, USA).

### 2.7 *In vitro* and *in vivo* systemic safety evaluation

Cell viability was then quantified using the Cell Counting Kit-8 (CCK-8) assay according to the manufacturer’s protocol, with optical density measured at 450 nm on a microplate reader. The results were normalized to the untreated control group (without H_2_O_2_ and without exosomes) and expressed as relative cell viability.

Animals were randomized into three experimental cohorts (five mice per group): a vehicle control (PBS) group, a BMSC-Exo treatment group, and a Cur-BMSC-Exo treatment group. Exosome preparations (200 μg in 200 μL PBS) or an equivalent volume of PBS were administered via the lateral tail vein every third day, yielding a cumulative total of four injections. Seven days following the final administration, retro-orbital blood samples were obtained under anesthesia, and serum was harvested by centrifugation at 3,000 × g for 15 min at 4 °C. Hepatic and renal function markers, including alanine aminotransferase (ALT), aspartate aminotransferase (AST), total bilirubin (TBIL), blood urea nitrogen (BUN), creatinine (CREA), and uric acid (UA), were quantified on a fully automated clinical chemistry analyzer (Roche, Switzerland).

For histopathological evaluation, mice were humanely euthanized and the heart, liver, spleen, lungs, and kidneys were promptly excised. Tissue specimens were fixed in 4% paraformaldehyde for 24 h, processed through graded alcohols and xylene, embedded in paraffin wax, and cut into 5-μm sections using a rotary microtome. The tissue slices were subjected to routine hematoxylin and eosin (H&E) staining and examined under an Olympus light microscope (Olympus, Japan) for morphological alterations.

### 2.8 Data analysis for bone single-cell RNA sequencing (scRNA-seq)

Single-cell RNA sequencing (scRNA-seq) was performed to profile the transcriptomic changes in bone marrow cells after treatment. Mice were randomly divided into two groups (n = 2 per group) and received four tail vein injections of either Cur-BMSC-Exo (200 μg in 200 μL PBS) or an equal volume of PBS every 72 h. One week after the last injection, femoral bone marrow cells were isolated for scRNA-seq library construction using the 10x Genomics Chromium platform, followed by sequencing on an Illumina NovaSeq 6000 system. Raw data processing, quality control, cell clustering, differential expression analysis, and functional enrichment were performed using standard bioinformatics pipelines. Detailed experimental and analytical procedures are provided in the Supplementary Material.

### 2.9 *In vitro* oxidative stress model and osteogenic differentiation assay

MC3T3-E1 pre-osteoblasts (subclone 14, ATCC) were maintained in α-MEM supplemented with 10% FBS and 1% penicillin-streptomycin. For validation in primary osteoblasts, calvarial osteoblasts were isolated from neonatal C57BL/6 mice (postnatal day 3) by sequential collagenase (0.1%) and trypsin (0.25%) digestion, and cultured in α-MEM with 10% FBS. An oxidative stress model was established by treating cells with 200 μM hydrogen peroxide for 2 h. Cells were then divided into six groups: (Black and Rosen, 2016) normal control (no H_2_O_2_); (Altkorn and Vokes, 2001) H_2_O_2_ + PBS; (Appelman-Dijkstra and Papapoulos, 2015) H_2_O_2_ + BMSC-Exo (100 μg/mL); (Huang et al., 2018) H_2_O_2_ + free curcumin (10 μM); (Tian et al., 2023) H_2_O_2_ + Cur-BMSC-Exo (100 μg/mL); and (Wang et al., 2023) H_2_O_2_ + Cur-BMSC-Exo + Mdivi-1 (10 μM, mitophagy inhibitor) (Yang et al., 2026). After 24 h of treatment, cells were cultured in osteogenic differentiation medium (α-MEM containing 10% FBS, 10 mM β-glycerophosphate, 50 μg/mL ascorbic acid, and 0.1 μM dexamethasone). Alkaline phosphatase (ALP) staining and Alizarin Red S (ARS) staining were performed on days 7 and 14 to evaluate early osteogenic differentiation and extracellular matrix mineralization, respectively.

For ALP quantification, the ALP-positive area was measured using ImageJ software (version 1.53k) with a consistent color threshold and expressed as a percentage of the total image area. For ARS, lower-magnification images capturing the entire well surface were acquired using a flatbed scanner to eliminate field-selection bias, and mineralized nodule area was quantified using ImageJ. Detailed experimental procedures are provided in the Supplementary Material.

### 2.10 Mitophagy detection

To investigate the effect of Cur-BMSC-Exos on mitochondrial function and autophagic activity, we assessed mitophagy flux by quantifying the colocalization of mitochondria (MitoTracker Green) and lysosomes (LysoTracker Red) using confocal laser scanning microscopy. Mitochondrial membrane potential, a key indicator of mitochondrial health, was determined using the JC-1 assay, which distinguishes between polarized (red fluorescence) and depolarized (green fluorescence) mitochondria. Western blot was performed for quantifying the relative protein expression of PINK1, PARKIN, P62 and LC3. All quantitative analyses were performed using ImageJ software. Full protocols are available in the Supplementary Material.

### 2.11 Ovariectomized (OVX) mouse model and treatment

A total of forty female C57BL/6 mice (12 weeks of age) were randomly allocated into five experimental cohorts (n = 8 per cohort): Sham-operated control mice receiving PBS; OVX mice receiving PBS alone; OVX mice receiving BMSC-Exo; OVX mice receiving Cur-BMSC-Exo; and OVX mice co-treated with Cur-BMSC-Exo plus Mdivi1 (Cassidy-Stone et al., 2008). Bilateral ovariectomy was conducted under isoflurane general anesthesia following a previously reported method. In brief, bilateral ovaries were accessed through a dorsal midline incision, double-ligated, and resected. Sham-operated controls underwent identical surgical exposure without ovarian removal and received PBS via tail vein injection on the same schedule as the OVX groups.

Four weeks post-surgery, therapeutic intervention commenced. Exosome suspensions (200 μg formulated in 200 μL PBS) or a matching volume of vehicle (PBS) were administered via tail vein injection at 3-day intervals, totaling eight doses over the treatment course. Concomitantly, Mdivi1 (25 mg/kg body weight) was delivered by intraperitoneal injection at 48-h intervals throughout the entire treatment window.

### 2.12 Micro-computed tomography (micro-CT) analysis

At the endpoint of the study (week 8 post-treatment initiation), mice were sacrificed and femoral bones were harvested, fixed in 4% PFA for 24 h, and stored in PBS until imaging. The fixed femora were analyzed by high-resolution micro-CT on a SkyScan 1,276 scanner (Bruker, Belgium) using the following acquisition parameters: 50 kV tube voltage, 200 μA tube current, 10 μm pixel resolution, and 0.5° rotational step. Tomographic reconstruction was carried out with NRecon software (version 1.7.4.2, Bruker). For trabecular morphometry, a cylindrical ROI positioned 0.5–1.5 mm distal to the femoral growth plate was selected, and the following parameters were computed using CTAn software (version 1.20.3.0, Bruker): BV/TV, Tb.Th, Tb.N, and Tb. Sp.

### 2.13 Histological and immunofluorescence staining

Following micro-CT analysis, femoral samples were decalcified, embedded in paraffin, and sectioned into 5-μm serial slices. Immunofluorescence staining was performed to detect the expression of osteocalcin (OCN), COL1A2, PINK1, and PARKIN, while immunohistochemistry was used to examine TOMM20 expression. The mean fluorescence intensity, number of immunopositive cells, and positive staining area were quantified using ImageJ software. Detailed experimental procedures are provided in the Supplementary Material.

### 2.14 Statistical analysis

Statistical computations were carried out with GraphPad Prism 9 (GraphPad Software, USA). Results are expressed as mean ± standard deviation (SD) derived from a minimum of three biologically independent experiments. Intergroup comparisons among multiple conditions were conducted by one-way analysis of variance (ANOVA) with Tukey’s multiple comparisons test as the post-hoc procedure. A threshold of P < 0.05 was set as the criterion for statistical significance. Significance levels are denoted as: *P < 0.05, **P < 0.01, ***P < 0.001, ****P < 0.0001; ns indicates not significant.

## 3 Results

### 3.1 Characterization of BMSCs and exosomes derived from untreated and curcumin-pretreated BMSCs

**FIGURE 1:**
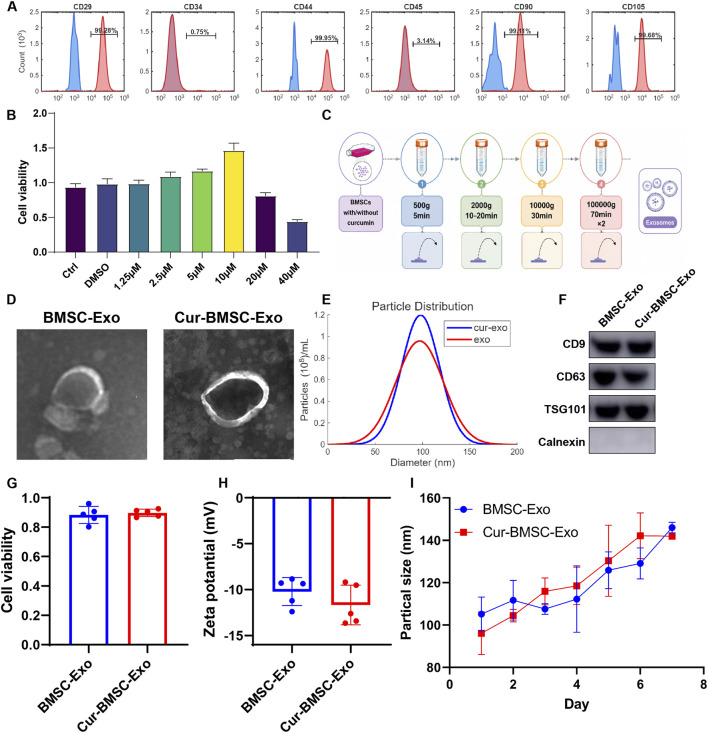
Characterization of BMSCs and exosomes derived from curcumin-pretreated BMSCs. **(A)** Flow cytometric analysis of BMSC surface markers. Isolated BMSCs were positive for mesenchymal markers CD29, CD44, CD90, and CD105, and negative for hematopoietic markers CD34 and CD45. **(B)** CCK-8 assay evaluating the effect of different curcumin concentrations (0–40 μM) on BMSC viability after 24 h incubation. 10 μM curcumin was selected as the optimal pretreatment concentration. **(C)** Schematic diagram illustrating the sequential differential ultracentrifugation protocol used for exosome isolation from BMSC conditioned medium. **(D)** Transmission electron microscopy (TEM) images showing the characteristic cup-shaped morphology of BMSC-Exo (left) and Cur-BMSC-Exo (right). Scale bar = 100 nm. **(E)** Nanoparticle tracking analysis (NTA) demonstrating the size distribution of BMSC-Exo (red) and Cur-BMSC-Exo (blue). **(F)** Western blotting analysis of exosome-specific markers (CD9, CD63, TSG101) and the negative control marker Calnexin in BMSC-Exo and Cur-BMSC-Exo. **(G)** Cell viability assay showing the absence of cytotoxicity of BMSC-Exo and Cur-BMSC-Exo (100 μg/mL) on normal target cells after 24 h treatment. **(H)** Zeta potential analysis of BMSC-Exo and Cur-BMSC-Exo measured by electrophoretic light scattering (ELS). Both preparations exhibited negative surface charge characteristic of mammalian cell-derived exosomes (ns, not significant between groups). **(I)** Storage stability assessment of BMSC-Exo and Cur-BMSC-Exo at 4 °C over 7 days, measured by changes in average particle size. All data are presented as mean ± SD. *p < 0.05, **p < 0.01, ***p < 0.001, ****p < 0.0001; ns, not significant.

Isolated bone marrow-derived mesenchymal stem cells (BMSCs) were first authenticated by flow cytometric analysis of standard surface markers. BMSCs exhibited high expression levels of mesenchymal lineage-specific markers CD29 (98.28%), CD44 (99.95%), CD90 (99.13%), and CD105 (99.68%), while showing negligible expression of hematopoietic markers CD34 (0.75%) and CD45 (3.14%), which is consistent with the internationally recognized immunophenotype of BMSCs (Figure 1A). To determine the non-toxic and effective concentration of curcumin for BMSC pretreatment, cell viability was assessed using the CCK-8 assay after 24-h incubation with curcumin at concentrations ranging from 0 to 40 μM. As depicted in Figure 1B, curcumin at concentrations of 1.25–10 μM was well-tolerated by BMSCs and even exerted a mild proliferative effect, with the maximum cell viability observed at 10 μM. In contrast, higher concentrations (20 μM and 40 μM) induced significant dose-dependent cytotoxicity. Therefore, 10 μM curcumin was selected as the preconditioning concentration for all subsequent experiments, based on cell viability rather than on osteogenic potency.

Exosomes were isolated from the conditioned media of untreated BMSCs and curcumin-pretreated BMSCs (Cur-BMSCs) using a standard sequential differential ultracentrifugation protocol, as illustrated in Figure 1C. Transmission electron microscopy (TEM) observation revealed that both BMSC-derived exosomes (BMSC-Exo) and Cur-BMSC-derived exosomes (Cur-BMSC-Exo) displayed the classic cup-shaped morphology with a distinct lipid bilayer membrane, which is the characteristic ultrastructure of exosomes (Figure 1D). Nanoparticle tracking analysis (NTA) was performed to determine the size distribution and concentration of the isolated exosomes. As shown in Figure 1E, both BMSC-Exo and Cur-BMSC-Exo had a narrow size distribution centered at approximately 100 nm, with the vast majority of particles falling within the 50–150 nm range typical of exosomes. Western blotting analysis further confirmed the exosomal identity and purity: both exosome preparations were strongly positive for the canonical exosome markers CD9, CD63, and TSG101, but completely negative for the endoplasmic reticulum marker Calnexin, indicating no contamination with cellular debris or apoptotic bodies (Figure 1F).

The biocompatibility of BMSC-Exo and Cur-BMSC-Exo was evaluated by treating normal target cells with exosomes at a concentration of 100 μg/mL for 24 h. As shown in Figure 1G, neither exosome preparation induced significant cytotoxicity, with cell viability remaining above 90% in both groups. To further characterize the physicochemical properties of the isolated exosomes, the surface charge was quantified by electrophoretic light scattering (ELS) using a Zetasizer Nano ZS system (Malvern Panalytical, United Kingdom). Both BMSC-Exo and Cur-BMSC-Exo exhibited negative zeta potentials, consistent with the characteristic surface charge of mammalian cell-derived exosomes. These results demonstrate that curcumin pretreatment did not substantially alter the surface charge characteristics or colloidal stability of the isolated exosomes (Figure 1H). The storage stability of exosomes was assessed by monitoring changes in average particle size over 7 days at 4 °C. NTA measurements showed no statistically significant between-group difference (BMSC-Exo versus Cur-BMSC-Exo) in average particle size from Day 0 to Day 7 (p > 0.05), and both size distributions remained within the canonical exosome range (Figure 1I).

### 3.2 *In vitro* cellular uptake, *in vivo* biodistribution and systemic safety evaluation of BMSC-Exo and Cur-BMSC-Exo

**FIGURE 2:**
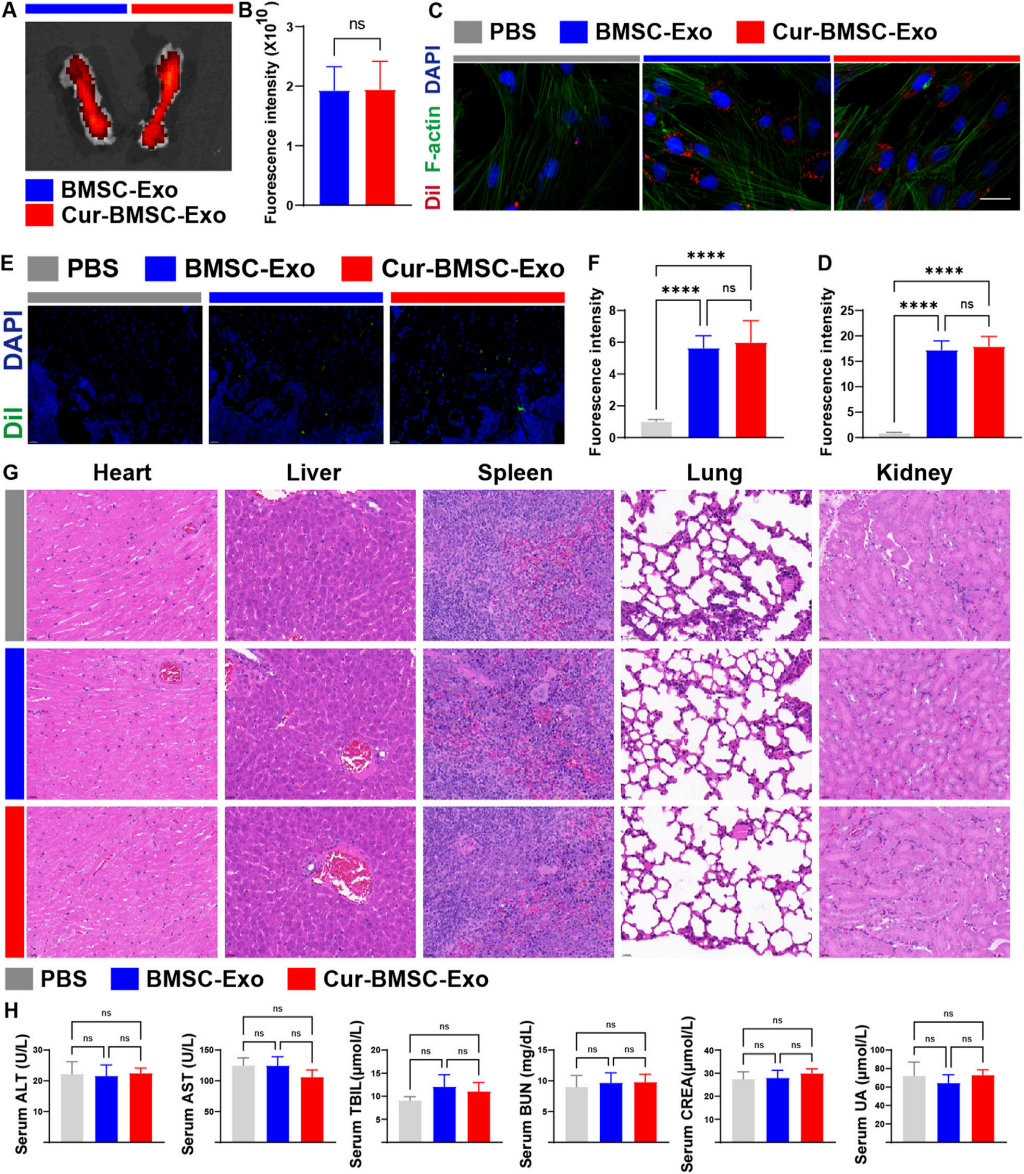
*In vitro* cellular uptake, *in vivo* biodistribution and systemic safety evaluation of BMSC-Exo and Cur-BMSC-Exo. **(A)** Representative *ex vivo* fluorescence images of excised femurs at 24 h after intravenous injection of DiI-labeled PBS, BMSC-Exo or Cur-BMSC-Exo. **(B)** Quantitative analysis of *ex vivo* fluorescence intensity in femoral tissues. **(C)** Confocal laser scanning microscopy images showing cellular uptake of DiI-labeled exosomes (red). Nuclei were stained with DAPI (blue) and cytoskeletons were stained with phalloidin (gray). Scale bar = 50 μm. **(D)** Quantitative analysis of fluorescence intensity from confocal images. **(E)** Fluorescence microscopy images of frozen femoral tissue sections at 24 h after intravenous injection of DiI-labeled PBS, BMSC-Exo or Cur-BMSC-Exo. Distinct DiI signals were observed throughout the bone tissue in mice treated with exosomes, but not in the PBS group. Scale bar = 100 μm. **(F)** Quantitative analysis of fluorescence intensity in frozen femoral tissue sections from *ex vivo* imaging. **(G)** Hematoxylin and eosin (H&E) staining of major organs (heart, liver, spleen, lung, kidney) from mice in PBS, BMSC-Exo and Cur-BMSC-Exo groups at 7 days post-injection. No obvious pathological abnormalities were observed in any group. Scale bar = 100 μm. **(H)** Serum biochemical parameters including ALT, AST, TBIL (liver function) and BUN, CREA, UA (renal function) in mice from different groups. All parameters showed no significant differences among the three groups. All data are presented as mean ± SD. *p < 0.05, **p < 0.01, ***p < 0.001, ****p < 0.0001; ns, not significant.

To investigate whether curcumin pretreatment alters the cellular internalization efficiency of BMSC-derived exosomes, Dil-labeled BMSC-Exo and Cur-BMSC-Exo were intravenously administered via the tail vein. *Ex vivo* fluorescence imaging of the excised femurs revealed robust Dil signals in both the BMSC-Exo and Cur-BMSC-Exo groups (Figure 2A). Quantitative analysis confirmed comparable fluorescence intensities between the two exosome-treated groups in femoral tissue, with no statistically significant difference (Figure 2B). This bone accumulation is attributable to the intrinsic bone-homing properties of BMSC-derived extracellular vesicles rather than to any curcumin payload, as the present study utilized exosomes derived from curcumin-pretreated BMSCs rather than exosomes loaded with curcumin.

We next assessed whether these exosomes could be internalized by osteoblast-lineage cells *in vitro*. BMSC cells were incubated with Dil-labeled BMSC-Exo or Cur-BMSC-Exo, and confocal microscopy revealed abundant intracellular Dil signals in the cytoplasm of cells treated with either formulation, whereas PBS-treated cells showed minimal background fluorescence (Figure 2C). Quantification of the mean fluorescence intensity demonstrated significantly elevated Dil signals in both exosome-treated groups compared to the PBS control (Figure 2D), with no significant difference between BMSC-Exo and Cur-BMSC-Exo, suggesting equivalent uptake efficiency by preosteoblasts.

To further validate the distribution of exosomes within bone tissue, frozen sections of the femurs were subjected to fluorescence microscopy. Distinct Dil signals were observed throughout the bone tissue in mice treated with BMSC-Exo and Cur-BMSC-Exo, but not in the PBS group (Figure 2E). Quantitative analysis of the fluorescence intensity showed significantly higher Dil signals in both exosome-treated groups compared to the control, with comparable levels between the two exosome formulations (Figure 2F). Collectively, these findings demonstrate that both BMSC-Exo and Cur-BMSC-Exo are readily internalized by osteoblast-lineage cells.

To evaluate the systemic safety of exosome administration, histopathological examination of major organs and serum biochemical analysis were performed 7 days after intravenous injection. Hematoxylin and eosin (H&E) staining revealed no obvious pathological changes, including inflammatory cell infiltration, tissue necrosis, or structural damage, in the heart, liver, spleen, lung, and kidney of mice in either exosome-treated group compared with the PBS control group (Figure 2G). Furthermore, serum biochemical parameters reflecting liver function (ALT, AST, TBIL) and renal function (BUN, CREA, UA) were all within the normal physiological range, and no statistically significant differences were observed among the three groups (Figure 2H, all ns, p > 0.05). Collectively, these data demonstrate that systemic administration of BMSC-Exo and Cur-BMSC-Exo does not induce obvious systemic toxicity in mice.

### 3.3 Single-cell transcriptomic analysis reveals Cur-BMSC-Exo promotes bone regeneration by modulating osteoblast-lineage cells

**FIGURE 3:**
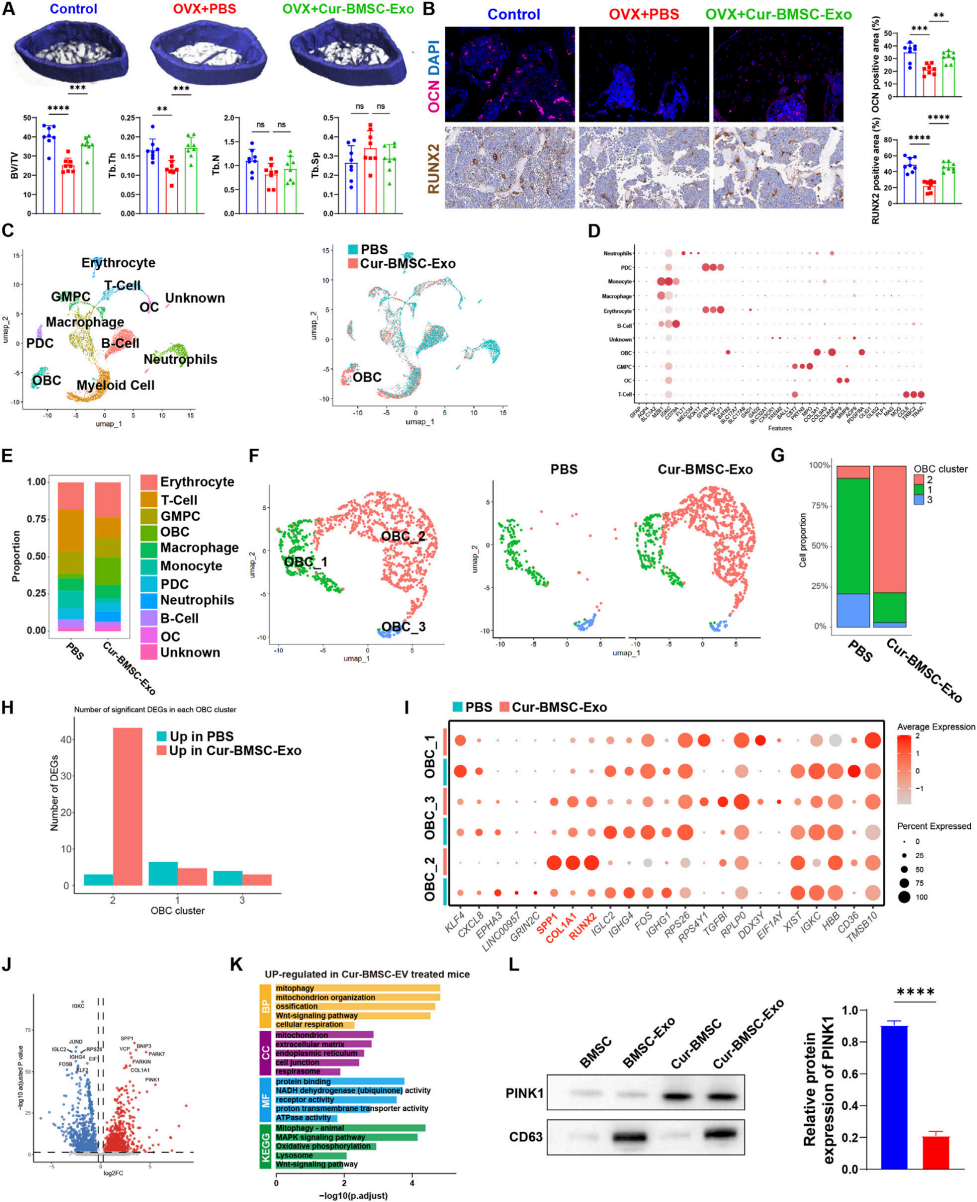
Single-cell transcriptomic analysis reveals Cur-BMSC-Exo promotes bone regeneration by modulating osteoblast-lineage cells. **(A)** Upper: Representative 3D micro-CT reconstructions of femoral trabecular bone from Sham operated group (Control), OVX + PBS, and OVX + Cur-BMSC-Exo mice. Lower: Quantitative analysis of trabecular bone parameters including bone volume/tissue volume (BV/TV), trabecular thickness (Tb.Th), trabecular number (Tb.N), and trabecular separation (Tb.Sp). **(B)** Upper: Representative immunofluorescence images of osteocalcin (OCN, magenta) staining in bone tissue sections; nuclei were counterstained with DAPI (blue). Lower: Representative immunohistochemical staining of RUNX2 (brown) in bone tissue sections. Right: Quantitative analysis of OCN-positive area (%) and RUNX2-positive area (%). Scale bar = 100 μm. **(C)** UMAP visualization of 11 distinct cell populations identified from mouse bone marrow cells, including erythrocytes, T cells, granulocyte-monocyte progenitor cells (GMPC), macrophages, monocytes (labeled as myeloid cells in panel C), plasmacytoid dendritic cells (PDC), B cells, osteoclasts (OC), neutrophils, osteoblast-lineage cells (OBCs), and unknown cells. **(D)** Dot plot showing the expression of canonical marker genes across the identified cell populations. **(E)** Left: Stacked bar plot showing the relative proportion of each cell type in PBS and Cur-BMSC-Exo groups. Right: Color legend for cell type annotation. **(F)** UMAP visualization of three transcriptionally distinct subpopulations of osteoblast-lineage cells (OBC_1, OBC_2, and OBC_3) in PBS and Cur-BMSC-Exo groups. **(G)** Stacked bar plot showing the proportion of each OBC subpopulation in PBS and Cur-BMSC-Exo groups. **(H)** Bar plot showing the number of differentially expressed genes (DEGs) in each OBC cluster that were significantly upregulated in PBS versus Cur-BMSC-Exo-treated mice. **(I)** Dot plot showing the expression levels of representative marker genes in the three OBC subpopulations from PBS and Cur-BMSC-Exo groups. Dot size represents the percentage of cells expressing the gene, and color intensity represents the average expression level. **(J)** Volcano plot showing differentially expressed genes in OBC_2 between Cur-BMSC-Exo and PBS groups. Red dots indicate significantly upregulated genes, and blue dots indicate significantly downregulated genes. Selected key genes are labeled. **(K)** Gene Ontology (GO) and Kyoto Encyclopedia of Genes and Genomes (KEGG) enrichment analysis of genes upregulated in OBC_2 from Cur-BMSC-Exo-treated mice. BP: Biological Process; CC: Cellular Component; MF: Molecular Function. **(L)** Representative Western blot images and quantification analysis showing PINK1 and CD63 expression in whole-cell lysates of BMSCs and curcumin-pretreated BMSCs (Cur-BMSC), as well as in purified exosomes (BMSC-Exo and Cur-BMSC-Exo). CD63 served as the exosomal loading control. All data are presented as mean ± SD. *p < 0.05, **p < 0.01, ***p < 0.001, ****p < 0.0001; ns, not significant.

To validate the pro-osteogenic effects of Cur-BMSC-Exo *in vivo*, we performed micro-CT analysis and immunofluorescence staining of bone tissues. Micro-CT results demonstrated that the OVX + PBS group exhibited marked osteoporotic changes characterized by significantly reduced BV/TV and Tb.Th compared with the Sham-operated control group, thereby confirming successful model establishment. By contrast, Cur-BMSC-Exo treatment significantly increased BV/TV and Tb.Th compared with the OVX + PBS group, while no significant differences were observed in trabecular number (Tb.N) and trabecular separation (Tb.Sp) (Figure 3A). Immunofluorescence staining for osteocalcin (OCN) and immunohistochemical staining for RUNX2 further confirmed that the expression of these osteogenic markers was significantly elevated in the Cur-BMSC-Exo group (Figure 3B), indicating increased bone formation following Cur-BMSC-Exo treatment.

To further identify the potential cellular targets of systemically administered Cur-BMSC-Exo within the heterogeneous bone marrow microenvironment in a discovery-driven manner, we performed exploratory single-cell RNA sequencing (scRNA-seq) prior to any targeted osteogenesis characterization. Unsupervised clustering and uniform manifold approximation and projection (UMAP) visualization identified 11 distinct cell populations based on canonical marker genes, including erythrocytes, T cells, granulocyte-monocyte progenitor cells (GMPC), macrophages, B cells, neutrophils, osteoblast-lineage cells (OBCs), and other immune cell subsets (Figures 3C,D). Cellular composition analysis revealed that Cur-BMSC-Exo treatment did not significantly alter the proportions of most immune and hematopoietic cell types. However, osteoblast-lineage cells (OBCs) exhibited the most pronounced transcriptional changes between the two groups, indicating that OBCs are the primary cellular target of Cur-BMSC-Exo *in vivo* (Figure 3E).

We further subclustered the OBC population and identified three transcriptionally distinct subpopulations, designated OBC_1, OBC_2, and OBC_3 (Figure 3F). Figure 3G illustrates the proportional distribution of each OBC subpopulation between the two conditions (PBS vs. Cur-BMSC-Exo). Comparative analysis of subpopulation proportions showed that Cur-BMSC-Exo treatment led to a shift in OBC subpopulation distribution, with a marked increase in the proportion of OBC_1 cells and a corresponding decrease in OBC_3 cells (Figure 3G). Differential gene expression (DEG) analysis was performed. OBC_2 was the most transcriptionally responsive subpopulation in Cur-BMSC-Exo-treated mice (Figure 3H). Dot plot analysis revealed that Cur-BMSC-Exo treatment significantly upregulated the expression of multiple key osteogenic genes in OBC_2, including Col1a1, Spp1, and Runx2, which are critical for osteoblast differentiation and extracellular matrix mineralization (Figure 3I). Volcano plot visualization further confirmed the global transcriptional upregulation of osteogenic-related genes in OBC_2 from Cur-BMSC-Exo-treated mice (Figure 3J). Gene Ontology (GO) and Kyoto Encyclopedia of Genes and Genomes (KEGG) enrichment analysis of the upregulated genes in Cur-BMSC-Exo-treated OBCs revealed significant enrichment in biological processes related to osteoblast differentiation, bone mineralization, mitochondrial organization, and cellular respiration. Pathway analysis further identified activation of the Wnt signaling pathway and MAPK signaling pathway, both of which are central regulators of bone formation (Figure 3K). Furthermore, quantitative Western blot analysis of exosome lysates revealed that Cur-BMSC-Exo contained significantly higher levels of PINK1 protein compared with BMSC-Exo, with densitometric quantification normalized to the exosomal loading control CD63 (Figure 3L), substantiating that curcumin preconditioning reprograms exosomal cargo composition.

### 3.4 Cur-BMSC-exo promotes osteogenic differentiation via activating mitophagy

**FIGURE 4:**
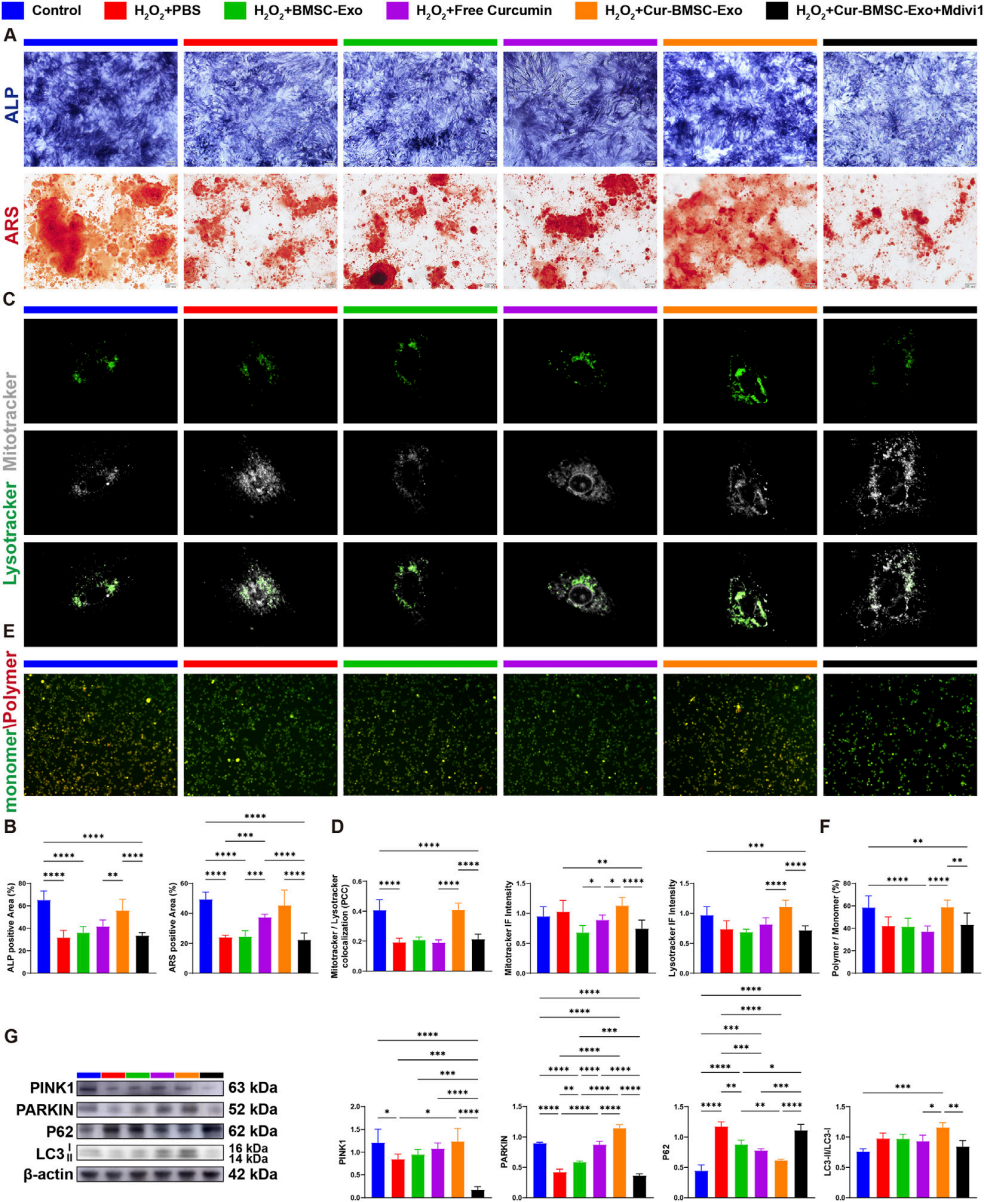
Cur-BMSC-Exo promotes osteogenic differentiation under oxidative stress via activating mitophagy. MC3T3-E1 pre-osteoblasts were divided into six groups: Control (no H_2_O_2_), H_2_O_2_ + PBS, H_2_O_2_ + BMSC-Exo, H_2_O_2_ + free curcumin, H_2_O_2_ + Cur-BMSC-Exo, and H_2_O_2_ + Cur-BMSC-Exo + Mdivi-1 (mitophagy inhibitor). **(A)** Alkaline phosphatase (ALP) staining (upper) and Alizarin Red S (ARS) staining (lower) showing osteogenic differentiation capacity. Lower-magnification images capturing the entire well surface are presented. Scale bar = 200 μm. **(B)** Quantitative analysis of ALP-positive area (%) and ARS-positive area (%). **(C)** Confocal laser scanning microscopy images showing MitoTracker (green, mitochondria), LysoTracker (red, lysosomes), and merged images. Merged images show colocalization of mitochondria and lysosomes (yellow/white), indicating mitophagy. Normal control cells displayed intact tubular mitochondrial networks and well-distributed punctate lysosomes with minimal baseline colocalization. H_2_O_2_-treated cells exhibited severe mitochondrial fragmentation and dispersed lysosomal distribution. Cur-BMSC-Exo restored elongated mitochondrial morphology and enhanced lysosomal clustering, whereas free curcumin showed only modest effects and Mdivi-1 co-treatment maintained mitochondrial fragmentation. Scale bar = 20 μm. **(D)** Quantitative analysis of mitochondria–lysosome Pearson colocalization coefficient (PCC), MitoTracker immunofluorescence intensity, and LysoTracker immunofluorescence intensity. **(E)** JC-1 staining showing mitochondrial membrane potential (MMP). Green fluorescence represents JC-1 monomers (low MMP), and red/yellow fluorescence represents JC-1 polymers (high MMP). All images were acquired under strictly identical confocal parameters to ensure quantitative comparability. Scale bar = 50 μm. **(F)** Quantitative analysis of the polymer/monomer fluorescence intensity ratio. **(G)** Representative Western blot images and quantification analyses showing the protein levels of PINK1 (63 kDa), Parkin (52 kDa), p62/SQSTM1 (62 kDa), LC3-I (16 kDa), and LC3-II (14 kDa) among groups. β-actin (42 kDa) was used as the loading control. All data are presented as mean ± SD. *p < 0.05, **p < 0.01, ***p < 0.001, ****p < 0.0001; ns, not significant.

To determine whether Cur-BMSC-Exo enhances osteogenesis through mitophagy in committed osteoblastic cells, MC3T3-E1 pre-osteoblasts were subjected to H_2_O_2_-induced oxidative stress and allocated to six groups: normal control (no H_2_O_2_), H_2_O_2_ + PBS, H_2_O_2_ + BMSC-Exo, H_2_O_2_ + free curcumin, H_2_O_2_ + Cur-BMSC-Exo, and H_2_O_2_ + Cur-BMSC-Exo + Mdivi-1. Hydrogen peroxide has been established as the primary ROS mediating osteoblast dysfunction in estrogen-deficient bone microenvironments (Chen S. et al., 2026; Zhang et al., 2026). Accordingly, exogenous H_2_O_2_ (200 μM) (Lee et al., 2015; Jin et al., 2020) was applied as a physiologically relevant oxidative challenge to recapitulate the mitochondrial redox imbalance and defective mitophagy characteristic of osteoblasts under postmenopausal conditions (Chen S. et al., 2026). H_2_O_2_ exposure markedly suppressed osteogenic differentiation, as shown by reduced ALP-positive area and ARS mineralization relative to the normal control (Figure 4A). Native BMSC-Exo partially rescued this impairment, whereas free curcumin alone produced only modest effects. Cur-BMSC-Exo significantly enhanced both early and late osteogenic differentiation, with ALP-positive and ARS-positive areas exceeding those of the H_2_O_2_ + PBS and H_2_O_2_ + BMSC-Exo groups. Co-treatment with Mdivi-1 abolished these Cur-BMSC-Exo-mediated benefits, indicating that functional mitophagy is essential for the observed pro-osteogenic effect (Figure 4B).

Mitophagy flux was visualized by dual staining of mitochondria (MitoTracker Green) and lysosomes (LysoTracker Red). Normal control cells displayed intact tubular mitochondrial networks and punctate lysosomes with minimal baseline colocalization. Following H_2_O_2_ treatment, cells exhibited severe mitochondrial fragmentation, dispersed lysosomal distribution, and a significantly decreased mitochondria–lysosome Pearson colocalization coefficient (PCC) (Figures 4C,D). Cur-BMSC-Exo restored elongated mitochondrial morphology, enhanced lysosomal clustering, and significantly increased the PCC compared with the H_2_O_2_ + PBS group, consistent with active mitophagy flux. Quantification further confirmed elevated MitoTracker and LysoTracker fluorescence intensities in the Cur-BMSC-Exo group (Figure 4D). Notably, free curcumin alone did not significantly activate mitophagy flux or restore mitochondrial morphology under oxidative stress. Mdivi-1 co-treatment reversed these Cur-BMSC-Exo-induced effects, maintaining mitochondrial fragmentation and reducing lysosomal recruitment. Mitochondrial membrane potential (MMP) was assessed using the JC-1 assay. H_2_O_2_ challenge caused a pronounced loss of MMP, reflected by a significantly reduced polymer-to-monomer fluorescence ratio. Cur-BMSC-Exo effectively restored MMP, achieving a polymer/monomer ratio significantly higher than those of the H_2_O_2_ + PBS, H_2_O_2_ + BMSC-Exo, and H_2_O_2_ + free curcumin groups (Figures 4E,F). Mdivi-1 co-treatment completely abrogated this restorative effect, resulting in sustained mitochondrial depolarization. All JC-1 images were acquired under strictly identical confocal parameters to ensure quantitative comparability. At the protein level, Cur-BMSC-Exo restored PINK1 and Parkin expression, reduced p62/SQSTM1 accumulation, and increased the LC3-II/LC3-I ratio compared with the H_2_O_2_ + PBS group; these effects were reduced by Mdivi-1 co-treatment (Figure 4G). Collectively, these data demonstrate that Cur-BMSC-Exo enhances osteogenic differentiation in osteoblast-lineage cells under oxidative stress by activating mitophagy and restoring mitochondrial function, and that this effect is strictly dependent on functional mitophagy and cannot be replicated by free curcumin alone.

### 3.5 Cur-BMSC-Exo ameliorates OVX-induced osteoporosis via activating PINK1/Parkin-mediated mitophagy *in vivo*

**FIGURE 5:**
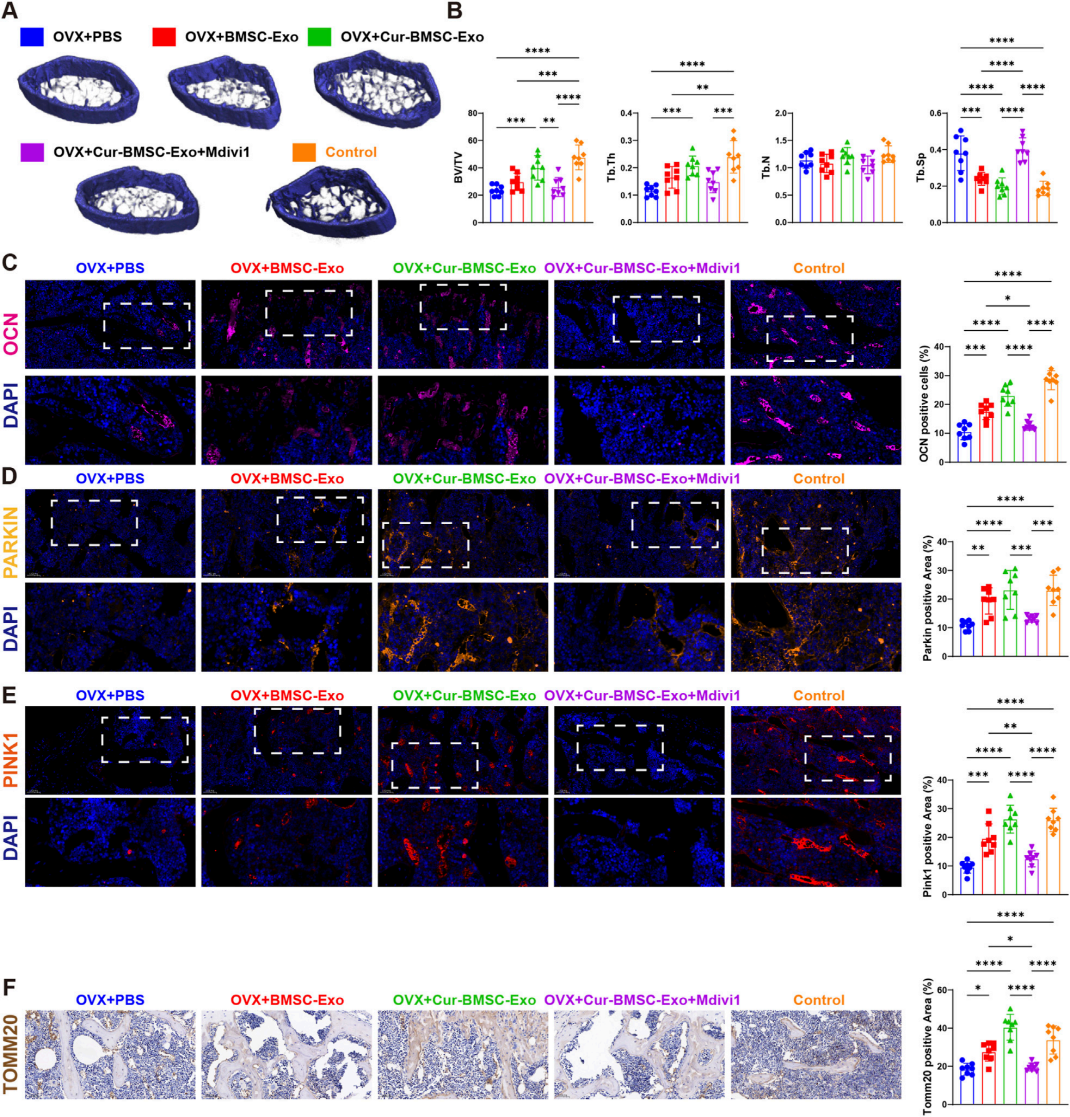
Cur-BMSC-Exo ameliorates OVX-induced osteoporosis via activating PINK1/Parkin-mediated mitophagy *in vivo*. OVX mice were randomized into five cohorts: Sham-operated control (Control), OVX + PBS, OVX + BMSC-Exo, OVX + Cur-BMSC-Exo, and OVX + Cur-BMSC-Exo + Mdivi-1. **(A)** Representative 3D micro-CT reconstructions of femoral trabecular bone from all experimental groups. **(B)** Quantitative analysis of trabecular bone parameters including bone volume/tissue volume (BV/TV), trabecular thickness (Tb.Th), trabecular number (Tb.N), and trabecular separation (Tb.Sp). **(C)** Immunofluorescence staining of osteocalcin (OCN, magenta), a mature osteoblast marker, in bone tissue sections from all experimental groups. Nuclei were counterstained with DAPI (blue). Dashed white boxes indicate regions of interest. Right panel: quantitative analysis of OCN-positive cells (%). **(D)** Immunofluorescence staining of PARKIN (orange) in bone tissue sections from all experimental groups. Nuclei were counterstained with DAPI (blue). Dashed white boxes indicate regions of interest. Right panel: quantitative analysis of PARKIN-positive area (%). **(E)** Immunofluorescence staining of PINK1 (red) in bone tissue sections from all experimental groups. Nuclei were counterstained with DAPI (blue). Dashed white boxes indicate regions of interest. Right panel: quantitative analysis of PINK1-positive area (%). **(F)** Immunohistochemical staining of TOMM20 (brown), a mitochondrial outer membrane protein, in bone tissue sections from all experimental groups. Right panel: quantitative analysis of TOMM20-positive area (%). All data are presented as mean ± SD. *p < 0.05, **p < 0.01, ***p < 0.001, ****p < 0.0001; ns, not significant.

To validate the therapeutic mechanism of Cur-BMSC-Exo in postmenopausal osteoporosis, ovariectomized (OVX) mice were randomized into five cohorts: Sham-operated control, OVX + PBS, OVX + BMSC-Exo, OVX + Cur-BMSC-Exo, and OVX + Cur-BMSC-Exo + Mdivi-1. Three-dimensional micro-CT analysis of femoral trabecular bone revealed intact trabecular architecture in Sham-operated mice, whereas the OVX + PBS group exhibited severe osteoporotic changes characterized by significantly reduced bone volume/tissue volume (BV/TV) and trabecular thickness (Tb.Th), together with increased trabecular separation (Tb.Sp), compared with the Sham group (Figures 5A,B). Native BMSC-Exo treatment partially attenuated these OVX-induced skeletal deficits. By contrast, Cur-BMSC-Exo conferred markedly superior protection, with BV/TV and Tb.Th significantly improved compared with the OVX + PBS group; however, these parameters were only partially restored and did not fully reach Sham levels, indicating incomplete reversal of OVX-induced bone loss. Co-administration of Mdivi-1 completely abolished the bone-protective effects of Cur-BMSC-Exo, yielding BV/TV, Tb.Th, and Tb. Sp comparable to the OVX + PBS group. No significant differences in trabecular number (Tb.N) were detected among the five groups. Immunofluorescence staining for osteocalcin (OCN), a specific marker of mature osteoblasts, showed that Cur-BMSC-Exo treatment significantly increased the proportion of OCN-positive cells in bone tissue compared with both the OVX + PBS and OVX + BMSC-Exo groups (Figure 5C). This pro-osteogenic effect was fully reversed by Mdivi-1 co-treatment, confirming that functional mitophagy is required for Cur-BMSC-Exo-induced bone formation *in vivo*.

To directly assess mitophagy activation, we examined the expression of key PINK1/Parkin pathway components. Immunofluorescence staining demonstrated that Cur-BMSC-Exo significantly upregulated PINK1 and PARKIN expression in bone marrow cells relative to the OVX + PBS group (Figures 5D,E). Concomitantly, TOMM20 expression was significantly reduced in the Cur-BMSC-Exo group, indicating enhanced clearance of damaged mitochondria (Figure 5F). In contrast, Mdivi-1 co-treatment completely blocked the upregulation of PINK1 and PARKIN, and restored TOMM20 expression to levels comparable to those in the OVX + PBS group. Collectively, these *in vivo* results provide definitive evidence that Cur-BMSC-Exo ameliorates OVX-induced osteoporosis by activating the PINK1/Parkin-mediated mitophagy pathway. Pharmacological inhibition of mitophagy with Mdivi-1 abolishes both the molecular and skeletal benefits of Cur-BMSC-Exo, further confirming that mitophagy is the central mechanism underlying its therapeutic efficacy.

## 4 Discussion

In this study, we systematically investigated the therapeutic potential and underlying mechanisms of curcumin-preconditioned bone marrow-derived mesenchymal stem cell exosomes (Cur-BMSC-Exo) for postmenopausal osteoporosis. Our major findings can be summarized as follows (Black and Rosen, 2016): Curcumin pretreatment at 10 μM significantly enhanced the osteogenic potential of BMSC exosomes without altering their basic physicochemical properties, cellular uptake efficiency, or *in vivo* biodistribution (Altkorn and Vokes, 2001); Single-cell RNA sequencing revealed that osteoblast-lineage cells (OBCs), are the primary cellular targets of Cur-BMSC-Exo *in vivo* (Appelman-Dijkstra and Papapoulos, 2015); Cur-BMSC-Exo exerted potent pro-osteogenic effects under oxidative stress conditions by activating PINK1/Parkin-mediated mitophagy, which was completely abrogated by the mitophagy inhibitor Mdivi1 (Huang et al., 2018); Systemic administration of Cur-BMSC-Exo significantly ameliorated ovariectomy (OVX)-induced bone loss in mice, providing a promising cell-free therapeutic strategy for postmenopausal osteoporosis.

Curcumin, a natural polyphenol derived from *C. longa*, possesses well-documented anti-inflammatory, antioxidant, and osteogenic properties that make it an attractive candidate for bone disease therapy (Kotha and Luthria, 2019; Sadeghi et al., 2023). However, its clinical translation has been severely hampered by its poor aqueous solubility (<1 μg/mL), low systemic bioavailability (<1%), and rapid metabolism (half-life <2 h) *in vivo* (Heidari et al., 2023). Preconditioning MSCs with curcumin has emerged as an innovative strategy to overcome these limitations (Serrenho et al., 2024), as it not only improves the intrinsic biological activity of MSCs but also endows their secreted exosomes with enhanced therapeutic potential through cargo reprogramming. Although previous studies have shown that direct curcumin loading into exosomes has shown efficacy across diverse pathologies, including renal ischemia-reperfusion injury (Chen B. et al., 2026), myocardial infarction (Chen et al., 2024), and wound healing (Li D. et al., 2023), its application to bone regeneration remains insufficiently explored. Our results further demonstrated that curcumin pretreatment induced global transcriptomic changes in BMSCs, leading to the enrichment of bioactive molecules related to antioxidant defense, mitochondrial quality control, and osteogenic differentiation in exosomes. Unlike direct drug loading approaches that often suffer from low encapsulation efficiency and burst release, curcumin preconditioning stimulates endogenous production of therapeutic cargoes by BMSCs, resulting in more stable and sustained biological effects. Notably, our data showed that curcumin pretreatment did not affect the size, morphology, surface marker expression, cellular uptake, or *in vivo* biodistribution of exosomes, indicating that the enhanced therapeutic efficacy of Cur-BMSC-Exo is primarily attributed to qualitative changes in their cargo composition rather than alterations in their pharmacokinetic properties.

Oxidative stress plays a central role in the pathogenesis of postmenopausal osteoporosis. Estrogen deficiency leads to excessive reactive oxygen species (ROS) production, which impairs osteoblast differentiation, induces osteoblast apoptosis, and disrupts the balance between bone formation and resorption (Iantomasi et al., 2023; Luo et al., 2025; Zhao et al., 2021). Mitochondria are the primary source of ROS in osteoblasts, and damaged mitochondria further exacerbate oxidative stress, creating a vicious cycle that accelerates bone loss (Palma et al., 2024; Zorov et al., 2014). Mitophagy, a selective form of autophagy that targets damaged mitochondria for degradation, is essential for maintaining mitochondrial function and cellular redox balance (Wang et al., 2020; Zeng et al., 2022; Yang et al., 2026). Emerging evidence suggests that dysregulated mitophagy contributes to the development of osteoporosis, and enhancing mitophagy may represent a novel therapeutic approach to promote bone formation (Ding et al., 2025; Guo et al., 2021; Hu et al., 2025; Li H. et al., 2024; Li W. et al., 2023). In this study, we demonstrated that Cur-BMSC-Exo treatment significantly increased the colocalization of mitochondria and lysosomes, restored mitochondrial membrane potential, and reduced ROS accumulation in H_2_O_2_-treated BMSCs. More importantly, the pro-osteogenic effects of Cur-BMSC-Exo were abolished by co-treatment with Mdivi1, providing direct causal evidence that mitophagy activation is essential for the therapeutic efficacy of Cur-BMSC-Exo. Furthermore, our *in vivo* results showed that Cur-BMSC-Exo treatment upregulated the expression of PINK1 and PARKIN (Eiyama and Okamoto, 2015; Li J. et al., 2023; Nguyen et al., 2016), key regulators of the canonical mitophagy pathway, and downregulated the expression of TOMM20, a mitochondrial outer membrane protein that is selectively degraded during mitophagy. These findings collectively indicate that Cur-BMSC-Exo ameliorates OVX-induced osteoporosis by activating the PINK1/Parkin-mediated mitophagy pathway, thereby clearing damaged mitochondria, breaking the oxidative stress vicious cycle, and promoting osteoblast differentiation.

A major strength of this study is the use of single-cell RNA sequencing to dissect the cellular and molecular mechanisms underlying the therapeutic effects of Cur-BMSC-Exo at single-cell resolution. Traditional bulk RNA sequencing approaches average gene expression across heterogeneous cell populations, making it difficult to identify the specific cell types targeted by therapeutic agents. In this study, we identified 11 distinct cell populations in mouse bone tissue and found that Cur-BMSC-Exo treatment did not significantly alter the proportions of most immune and hematopoietic cell types. Instead, osteoblast-lineage cells exhibited the most pronounced transcriptional changes, indicating that they are the primary cellular targets of Cur-BMSC-Exo *in vivo*. Furthermore, we subclustered the OBC population into three transcriptionally distinct subpopulations and found that OBC_2 was the most responsive subpopulation to Cur-BMSC-Exo treatment, with significant upregulation of key osteogenic genes such as *Col1a1*, *Spp1*, *Runx2*. These findings not only provide novel insights into the cell-specific effects of Cur-BMSC-Exo but also identify the OBC_2 subpopulation as a potential therapeutic target for bone regeneration.

Beyond the curcumin-based preconditioning strategy described herein, emerging exosome engineering and preconditioning approaches have demonstrated considerable promise in bone regenerative medicine. Notably, lithium-based preconditioning represents a particularly relevant paradigm, as lithium ions function as classical autophagy modulators (Motoi et al., 2014; Puglisi-Allegra et al., 2023) that concurrently activate osteogenic signaling pathways (Wang et al., 2024). In models of glucocorticoid-induced osteonecrosis of the femoral head (GC-ONFH), BMSCs stimulated by lithium ions secrete exosomes (Li-Exo) that exhibit enhanced immunomodulatory, osteogenic, and angiogenic functionalities. Specifically, Li-Exo promotes macrophage M2 polarization while suppressing M1 polarization, thereby creating a pro-regenerative inflammatory milieu (Chen et al., 2023). Furthermore, lithium-substituted bioglass ceramics (Li-BGC) can indirectly potentiate tissue repair by reprogramming the exosomal cargo of BMSCs (Liu et al., 2023); for example, Li-BGC treatment upregulates exosomal miR-455-3p, which suppresses HDAC2 and enhances histone H3 acetylation to promote chondrogenesis. Collectively, these studies establish that lithium-based preconditioning enhances exosome functionality through autophagy modulation, immunomodulatory reprogramming, and pro-angiogenic cargo enrichment, offering a mechanistically complementary framework to the PINK1/Parkin-mediated mitophagy activation conferred by curcumin preconditioning.

In parallel, metal ion stimulation and biomaterial-assisted delivery systems represent additional strategies to improve exosome yield, retention, and localized functionality. Copper-lithium-doped nanohydroxyapatite (Cu-Li-nHA) scaffolds, fabricated via gas foaming, promote MSC migration and homing through upregulation of the HIF-1α/SDF-1 pathway, thereby establishing a regenerative microenvironment that supports both osteogenesis and angiogenesis in osteonecrotic lesions (Li et al., 2022). Concurrently, extracellular matrix (ECM)-mimicking hydrogels, such as methacryloylated type I collagen-based Lightgel, serve as injectable depots that enable sustained local release of exosomes, markedly improving *in vivo* retention and therapeutic efficacy compared with systemic bolus administration (Chen et al., 2023). Thus, the integration of pharmacological preconditioning (e.g., curcumin or lithium) with biomaterial-based delivery platforms may represent an optimal future direction, combining cargo-mediated mechanistic specificity with enhanced local retention and controlled release kinetics.

The therapeutic potential of mesenchymal stem cell (MSC)-derived exosomes can be significantly enhanced through various preconditioning and bioengineering strategies. Preconditioning strategies, such as hypoxia, cytokine exposure, and pharmacological agents, have been shown to improve the yield and therapeutic efficacy of MSC-derived exosomes. For instance, TNF-α preconditioning of infrapatellar fat pad MSCs has been demonstrated to enhance exosome secretion and therapeutic efficacy in osteoarthritis models by activating the PI3K/AKT signaling pathway and upregulating autophagy-related proteins (Wu et al., 2024). Similarly, hypoxic preconditioning has been reported to boost the antioxidant and anti-apoptotic capacities of MSC-derived exosomes, primarily through the upregulation of specific microRNAs like miR-486-5p, which modulate key signaling pathways such as the PI3K/Akt axis (Zeng et al., 2025). Furthermore, bioengineering techniques have been employed to modify MSC-derived exosomes for enhanced therapeutic outcomes. For example, exosomes can be engineered to carry specific therapeutic molecules, such as microRNAs, to target disease-specific pathways. In the context of myocardial infarction, MSC-derived exosomes pretreated with vericiguat have been shown to enhance cardioprotective effects through the delivery of miR-1180-3p, which targets the ETS1 signaling pathway to reduce cardiac fibrosis (Li et al., 2025). Similarly, exosomes engineered to deliver miR-210 have demonstrated protective effects in renal ischemia-reperfusion injury models by improving cell survival and reducing oxidative stress (Toghiani et al., 2024). While these approaches each offer distinct advantages, our study specifically positions curcumin preconditioning as a clinically attractive strategy that not only enhances exosome bioactivity but also confers precise mechanistic specificity for activating of PINK1/Parkin-mediated mitophagy in osteoblast-lineage cells. This mitophagy-targeted mechanism distinguishes Cur-BMSC-Exo from other preconditioning paradigms and highlights the value of tailoring exosome engineering strategies to address mitochondrial dysfunction and oxidative stress in postmenopausal osteoporosis.

Despite the promising results, this study has several limitations that need to be addressed in future research. First, we did not identify the specific bioactive cargoes in Cur-BMSC-Exo that are responsible for activating mitophagy and promoting osteogenesis. Previous studies have shown that curcumin-preconditioned exosomes are enriched with various microRNAs, proteins, and lipids that can regulate mitochondrial function and autophagy. For example, in a recent study on renal ischemia-reperfusion injury, curcumin was found to increase the expression of miR-16-5p in BMSC exosomes by regulating CYP1B1 activity, which in turn inhibited ferroptosis by targeting the Smad3/Mb axis. Future studies should integrate transcriptomics, proteomics, and lipidomics to comprehensively characterize the cargo differences between BMSC-Exo and Cur-BMSC-Exo and identify the key molecules mediating their therapeutic effects. Second, although Mdivi1 is widely used as a specific inhibitor of mitophagy, it has been reported to have off-target effects on other cellular processes. To further confirm the role of mitophagy in the therapeutic effects of Cur-BMSC-Exo, future studies should use genetic approaches such as PINK1 or Parkin knockout mice. Third, the single-cell RNA sequencing was performed as an exploratory, hypothesis-generating screen with two biological replicates per group due to budgetary constraints, which inherently limits the statistical power for detecting subtle transcriptional changes and inter-individual variability. Nevertheless, this sample size is consistent with established practices for exploratory scRNA-seq analyses aimed at identifying major cell-population shifts and pathway enrichments. Importantly, all key mechanistic conclusions derived from the scRNA-seq dataset, including the identification of osteoblast-lineage cells as the primary transcriptional responders and the enrichment of mitophagy-related pathways, have been robustly validated through multiple independent experimental modalities with adequate statistical power, including *in vitro* osteogenic differentiation assays, mitophagy flux and mitochondrial function analyses, and *in vivo* micro-CT and histological evaluations. Future studies should increase the biological replicate number to enhance statistical power and reduce inter-individual variability. Fourth, this study was conducted in a mouse model of postmenopausal osteoporosis, and the results may not be directly translatable to humans. Future studies should validate the therapeutic efficacy of Cur-BMSC-Exo in large animal models and eventually in clinical trials. Finally, the long-term safety of systemic exosome administration needs to be thoroughly evaluated before clinical application, although our preliminary results showed no obvious systemic toxicity in mice.

In conclusion, this study demonstrates that curcumin-preconditioned BMSC exosomes exert potent therapeutic effects on postmenopausal osteoporosis by activating PINK1/Parkin-mediated mitophagy in osteoblast-lineage cells. Curcumin pretreatment enhances the osteogenic potential of BMSC exosomes by modulating their cargo content, without altering their basic properties or biodistribution. Single-cell RNA sequencing further identifies osteoblast-lineage cells, particularly the OBC_2 subpopulation, as the primary cellular targets of Cur-BMSC-Exo. These findings not only provide novel insights into the mechanism of action of Cur-BMSC-Exo but also support its potential as a safe and effective cell-free therapeutic strategy for postmenopausal osteoporosis. Future studies should focus on identifying the key therapeutic cargoes in Cur-BMSC-Exo, optimizing their production and delivery, and evaluating their long-term safety and efficacy in clinical settings.

## Data Availability

The original contributions presented in the study are included in the article/Supplementary Material, further inquiries can be directed to the corresponding authors.
