# Supplementary material for: Curcumin-preconditioned bone marrow mesenchymal stem cell exosomes ameliorate postmenopausal osteoporosis by activating mitophagy in osteoblast-lineage cells
Source: Front Cell Dev Biol. 2026 Jul 29;14:1891247. doi: 10.3389/fcell.2026.1891247 (PMC13461600; doi:10.3389/fcell.2026.1891247)
Supplement: Supplementary file 1 [file DataSheet1.docx]

**Isolation and Characterization of BMSC-Derived Exosomes**

Exosome purification was carried out by differential ultracentrifugation of conditioned medium harvested from BMSCs with or without curcumin preconditioning (Cur-BMSCs). Upon reaching approximately 80% confluence, the cells were switched to fresh medium supplemented with exosome-depleted FBS (System Biosciences, USA) and maintained for a further 48 h. The resulting conditioned medium was subjected to a stepwise centrifugation sequence — 500 × g for 5 min, 2,000 × g for 10 min, and 10,000 × g for 30 min at 4°C — to pellet cells, cellular debris, and larger membrane vesicles. The clarified supernatant was then transferred to ultracentrifuge tubes and spun at 100,000 × g for 70 min at 4°C in a Beckman Coulter Optima XE-90 rotor (Beckman Coulter, USA). The crude pellet was gently redissolved in ice-cold phosphate-buffered saline (PBS) and subjected to a second round of ultracentrifugation under identical conditions (100,000 × g, 70 min, 4°C). The final pellet, enriched in exosomes, was suspended in PBS and snap-frozen at −80°C pending downstream applications. Exosomal protein content was quantified with a BCA assay kit (Thermo Fisher Scientific, USA).

For immunoblotting of exosomal markers, 20 μg of exosome lysate was loaded onto 10% SDS-PAGE gels, separated by electrophoresis, and transferred to PVDF membranes (Millipore, USA). The membranes were then blocked with 5% non-fat dry milk in TBST for 60 min at room temperature, followed by overnight incubation at 4°C with primary antibodies specific for CD9, CD63, TSG101, and Calnexin (all at 1:1000 dilution; Abcam, ab2215, ab134045, ab125011, and ab22595, respectively). Subsequently, the membranes were washed with TBST and exposed to HRP-conjugated secondary antibodies (1:5000; Cell Signaling Technology, USA) for 1 h at room temperature. Signal detection was performed using an ECL reagent kit (Thermo Fisher Scientific, USA), and band images were acquired with a ChemiDoc XRS+ system (Bio-Rad, USA).

**In Vitro Cellular Uptake of Exosomes**

The lipophilic fluorophore DiI (Invitrogen, USA) was used to label exosomal membranes according to the supplier's protocol. Briefly, 5 μL of 1 mM DiI working solution was added to 100 μg of exosomal protein, and the mixture was incubated at 37°C for 30 minutes in the dark. To remove excess free dye, the exosome suspension was subjected to ultracentrifugation at 100,000 × g for 70 minutes at 4°C. The pelleted DiI-labeled exosomes were resuspended in PBS and stored at 4°C prior to use.

Flow cytometry was employed to quantify exosome uptake by BMSCs. Briefly, BMSCs were plated in 6-well plates at 1 × 10⁵ cells per well and incubated with 100 μg/mL DiI-labeled BMSC-Exo or Cur-BMSC-Exo for 6 hours. Cells were then collected, washed twice with ice-cold PBS, and analyzed on a BD FACSCanto II flow cytometer.

For confocal imaging analysis, BMSCs were seeded onto glass coverslips in 24-well plates at 5 × 10⁴ cells per well. After 24 hours of culture, cells were incubated with DiI-labeled exosomes for 6 hours. Following incubation, cells were fixed with 4% paraformaldehyde for 15 minutes, permeabilized with 0.1% Triton X-100 for 10 minutes, and stained with FITC-conjugated phalloidin (1:200; Sigma-Aldrich, USA) for 30 minutes to label F-actin. Nuclei were counterstained with DAPI (1:1000; Sigma-Aldrich) for 5 minutes. Coverslips were then mounted on slides and observed under a Zeiss LSM 880 confocal microscope.

**Single-Cell RNA Sequencing (scRNA-seq) and Data Analysis**

For single-cell transcriptomic profiling, mice were assigned to two cohorts (n = 2 each): a PBS vehicle group and a Cur-BMSC-Exo group. Treatments, either exosomes (200 μg in 200 μL PBS) or an equal volume of PBS, were delivered by tail vein injection every 72 h for four cumulative doses. One week after the final injection, animals were euthanized and femoral bones were dissected. Marrow was flushed from the femoral shafts with α-MEM, and the resulting cell suspensions were passed through a 70-μm nylon filter to obtain single-cell preparations. scRNA-seq libraries were prepared with the 10x Genomics Chromium Single Cell 3′ Reagent Kit v3.1 (10x Genomics, USA) following the supplier's protocol. Paired-end sequencing (150 bp) was carried out on an Illumina NovaSeq 6000 instrument (Illumina, USA).

Raw reads were demultiplexed and aligned using Cell Ranger v6.1.2 (10x Genomics) to produce gene-cell count matrices. Downstream analysis was conducted in R with Seurat v4.3.0. Quality-control filtering excluded cells expressing fewer than 200 genes, more than 6,000 genes, or with mitochondrial transcript fractions exceeding 10%, thereby eliminating debris, dead cells, and potential doublets. The filtered dataset was normalized, scaled, and dimensionally reduced by PCA. Unsupervised graph-based clustering was executed with FindClusters (resolution = 0.5), and UMAP was employed for two-dimensional visualization. Cluster identity was assigned by mapping canonical lineage-specific marker gene expression.

Intergroup differential expression analysis between PBS and Cur-MSC-EXO conditions was implemented with FindMarkers, applying thresholds of |log₂ fold change| > 0.25 and adjusted P value < 0.05. Enrichment analysis of Gene Ontology (GO) terms and Kyoto Encyclopedia of Genes and Genomes (KEGG) pathways was carried out on differentially expressed genes using clusterProfiler v4.6.2.

**In Vitro Oxidative Stress Model and Osteogenic Differentiation Assay**

MC3T3-E1 pre-osteoblasts (subclone 14, ATCC) were maintained in α-MEM supplemented with 10% FBS and 1% penicillin-streptomycin. An oxidative stress model was then established by exposing the cultures to 200 μM hydrogen peroxide (H₂O₂; Sigma-Aldrich, USA) for 2 h. Following preconditioning, cells were distributed into four experimental groups : (i) H₂O₂ plus PBS vehicle; (ii) H₂O₂ plus BMSC-derived exosomes (100 μg/mL); (iii) H₂O₂ plus curcumin-pretreated BMSC-derived exosomes (100 μg/mL); and (iv) H₂O₂ plus Cur-BMSC-Exo (100 μg/mL) in combination with 10 μM Mdivi1 (a selective mitophagy inhibitor; Sigma-Aldrich, USA).

Following a 24-h incubation with the respective treatments, the culture medium was exchanged for osteogenic differentiation medium consisting of α-MEM, 10% FBS, 10 mM β-glycerophosphate, 50 μg/mL L-ascorbic acid, and 100 nM dexamethasone. Fresh osteogenic medium was replenished every 72 h thereafter.

Following 7 days of osteogenic differentiation, alkaline phosphatase (ALP) activity was assessed using a commercial ALP staining kit (Beyotime, China) per the supplier's protocol. Cultures were immersion-fixed in 4% paraformaldehyde for 15 min and subsequently reacted with the ALP chromogenic working solution for 30 min at 37°C under light-protected conditions. Positively stained cells were examined by bright-field microscopy, and ALP-positive cell counts were enumerated using ImageJ software (version 1.53t; NIH, USA).

Following two weeks of osteogenic differentiation, extracellular matrix mineralization was assessed via Alizarin Red S staining. Cell monolayers were preserved in 4% PFA for 15 minutes, then exposed to 1% alizarin red S solution (pH 4.2, Sigma-Aldrich, USA) for 30 minutes at room temperature. Stained cultures were visualized under a light microscope, and the mineralized area was measured and analyzed with ImageJ software.

**Mitophagy Detection**

For evaluation of mitophagy flux, BMSCs were plated onto glass coverslips placed in 24-well plates at a density of 5×10⁴ cells/well and subjected to the aforementioned treatments. Twenty-four hours later, living cells were loaded with 200 nM MitoTracker Green FM and 50 nM LysoTracker Red DND-99 (both Invitrogen, USA) for 30 min at 37°C under light-protected conditions. Following live-cell staining, specimens were fixed with 4% paraformaldehyde for 15 min, and nuclear DNA was labeled with DAPI for 5 min. Coverslips were then mounted onto glass slides and examined under a Zeiss LSM 880 confocal microscope (Zeiss, Germany). The degree of mitochondrial-lysosomal colocalization was quantified by calculating the colocalization coefficient with ImageJ software.

To assess mitochondrial membrane potential (ΔΨm), we employed a JC-1 assay kit (Beyotime, China) following the supplier's protocol. BMSCs were seeded in 6-well plates at 1×10⁵ cells/well and exposed to the designated treatments. After 24 h, the cultures were incubated with JC-1 working reagent for 20 min at 37°C in the absence of light. Cells were subsequently rinsed twice with JC-1 assay buffer and imaged on a Zeiss LSM 880 confocal microscope. The relative MMP level was expressed as the ratio of red-emission JC-1 aggregates (indicating polarized, high ΔΨm) to green-emission JC-1 monomers (indicating depolarized, low ΔΨm), quantified using ImageJ.

For Western blot analysis, the following primary antibodies were used at the indicated dilutions: PINK1 (1:1000), Parkin (1:1000), p62/SQSTM1 (1:1000), LC3B (1:1000), and β-actin (1:5000). Horseradish peroxidase (HRP)-conjugated secondary antibodies were applied at 1:5000 dilution. Protein bands were visualized using an enhanced chemiluminescence (ECL) detection system.

**Histological and Immunofluorescence Staining**

Following μCT acquisition, femoral specimens were demineralized in 10% EDTA (pH 7.4) at 4°C for two weeks, then processed through graded alcohols, cleared in xylene, infiltrated with paraffin, and cut into 5-μm serial sections.

For immunofluorescence labeling, tissue sections were dewaxed, rehydrated through descending ethanol series, and subjected to heat-mediated antigen retrieval in 10 mM sodium citrate buffer (pH 6.0) for 15 min. Nonspecific binding sites were blocked with 5% bovine serum albumin (BSA) for 1 h at ambient temperature, followed by overnight incubation at 4°C with the following primary antibodies: anti-osteocalcin (OCN; 1:200; Abcam, ab13420), anti-PINK1 (1:200; Abcam, ab23707), anti-PARKIN (1:200; Abcam, ab77924), and anti-COL1A2 (1:200; Abcam, ab96723). After three washes in PBS, sections were reacted with Alexa Fluor 594-conjugated secondary antibodies (1:500; Invitrogen, USA) for 1 h at room temperature protected from light. Nuclear counterstaining was performed with DAPI (5 min). Slides were then examined under a Zeiss LSM 880 confocal microscope, and both mean fluorescence intensity and the number of immunopositive cells were quantified with ImageJ.

For immunohistochemical detection of TOMM20, paraffin-embedded sections were dewaxed, rehydrated, and antigen-retrieved as above. Endogenous peroxidase was inactivated by incubation with 3% hydrogen peroxide for 10 min. Sections were then blocked with 5% BSA (1 h, room temperature) and incubated overnight at 4°C with anti-TOMM20 antibody (1:200; Abcam, ab186735). Following PBS washes, HRP-conjugated secondary antibodies were applied for 1 h at room temperature. Immunoreactivity was developed with a DAB chromogen kit (Vector Laboratories, USA), and nuclei were counterstained with hematoxylin. The percentage of TOMM20-positive immunostaining area was measured using ImageJ.
